# Carbon Dots/Iron Oxide Nanoparticles with Tuneable Composition and Properties

**DOI:** 10.3390/nano12040674

**Published:** 2022-02-17

**Authors:** Joanna D. Stachowska, Monika B. Gamża, Claire Mellor, Ella N. Gibbons, Marta J. Krysmann, Antonios Kelarakis, Elżbieta Gumieniczek-Chłopek, Tomasz Strączek, Czesław Kapusta, Anna Szwajca

**Affiliations:** 1 School of Dentistry, University of Central Lancashire, Preston PR1 2HE, UK; jstachowska@uclan.ac.uk (J.D.S.); engibbons3@uclan.ac.uk (E.N.G.); mkrysmann@uclan.ac.uk (M.J.K.); 2 Jeremiah Horrocks Institute for Mathematics, Physics, and Astrophysics, University of Central Lancashire, Preston PR1 2HE, UK; mgamza@uclan.ac.uk; 3 UCLan Research Centre for Smart Materials, School of Natural Sciences, University of Central Lancashire, Preston PR1 2HE, UK; 4 School of Phycology and Computer Science, University of Central Lancashire, Preston PR1 2HE, UK; cmellor3@uclan.ac.uk; 5 Faculty of Physics and Applied Computer Science, AGH University of Science and Technology, Mickiewicza Ave. 30, 30-059 Krakow, Poland; echlopek@agh.edu.pl (E.G.-C.); tomasz.straczek@fis.agh.edu.pl (T.S.); kapusta@agh.edu.pl (C.K.); 6 Faculty of Chemistry, Adam Mickiewicz University, Umultowska 89b, 61-614 Poznań, Poland; anna.szwajca@amu.edu.pl

**Keywords:** magnetization, photoluminescence, carbon dots, magnetic nanoparticles, antimicrobial

## Abstract

We present a simple strategy to generate a family of carbon dots/iron oxide nanoparticles (C/Fe-NPs) that relies on the thermal decomposition of iron (III) acetylacetonate in the presence of a highly fluorescent carbon-rich precursor (derived via thermal treatment of ethanolamine and citric acid at 180 °C), while polyethylene glycol serves as the passivation agent. By varying the molar ratio of the reactants, a series of C/Fe-NPs have been synthesized with tuneable elemental composition in terms of C, H, O, N and Fe. The quantum yield is enhanced from 6 to 9% as the carbon content increases from 27 to 36 wt%, while the room temperature saturation magnetization is improved from 4.1 to 17.7 emu/g as the iron content is enriched from 17 to 31 wt%. In addition, the C/Fe-NPs show excellent antimicrobial properties, minimal cytotoxicity and demonstrate promising bioimaging capabilities, thus showing great potential for the development of advanced diagnostic tools.

## 1. Introduction

Superparamagnetic iron oxide nanoparticles (Fe-NPs) with sizes below 20 nm have been widely explored in a wide range of biomedical applications, including magnetically controlled gene and drug delivery [1,2], hyperthermia treatment [3], bioseparation of cells [4], cell tracking and tissue repair [1], magnetic resonance imaging (MRI) [5,6], biosensors [7], diagnosis and treatment of bacterial infections [8]. Common strategies for the preparation of Fe-NPs rely on thermal decomposition [9], coprecipitation [10,11], sol-gel methods [12], hydrothermal and solvothermal treatment of iron-rich precursors [5]. Accurate control of the reaction conditions (nature of organometallic reactants, iron salts, surfactants, solvents, temperature, duration), purification protocols and passivation strategies can lead to tailor-made Fe-NPs with narrow size distribution [13].

Given that Fe-NPs are susceptible to oxidation and are particularly prone to agglomeration, surface protection and passivation strategies are essential to impart long-term structural and colloidal stability. To that end, surfactants and macromolecules are physically adsorbed or chemically attached to Fe-NPs, while passivating layers based on precious metals, silica and carbon can accommodate various functional groups, facilitating further conjugation with ligands, biomolecules and nanostructures. An expanding body of literature focuses on Fe-NPs combined with peptides [14], DNA [15], aptamers [16], lipids [17], drug molecules [18], biopolymers [19], dendrimers [20], rare earth elements [21], metal organic frameworks [22], graphene oxide [23], carbon nanotubes [24] and fullerenes [25].

Particular emphasis is given to the development of hybrid materials based on Fe-NPs that exhibit fluorescent properties stemming from the presence of organic dyes [26], quantum dots [27], graphene quantum dots [28] and carbon dots (C-dots) [29]. Heteroatom doping can alter the electronic and optical properties of C-dots, oftentimes giving rise to red-shifted emissive systems [30]. In particular, hybrid nanoparticles composed of Fe-NPs and C-dots (C/Fe-NPs) combine the supreme magnetic properties of Fe-NPs with the excellent photoluminescent behavior and non-toxic nature of C-dots [31,32] and are ideal candidates for photothermal therapy [33], optical bioimaging [34], dual-modal bioimaging [35], bacteria sensing [36] and antimicrobial treatment [37]. Although, a number of interesting approaches have been proposed for the synthesis of C/Fe-NPs [16,29,36,38,39,40,41,42,43,44], the development of environmentally benign, cost and time effective synthetic strategies yielding a versatile range of C/Fe-NPs remains an open challenge.

In this study we demonstrate a facile method to fine-tune the chemical composition and, thereby, the optical and magnetic properties of C/Fe-NPs, thus generating a novel family of multifunctional nanomaterials. We present a simple strategy to synthesize a series of C/Fe-NPs with varying chemical composition that exhibit tuneable magnetization coupled with superior wavelength-dependent optical properties, bioimaging capabilities, non-toxic nature and antimicrobial activity.

## 2. Materials and Methods

### 2.1. Materials

Iron (III) acetylacetonate ≥99.9% trace metal basis (Fe(acac)_3_) and ethanolamine ≥98% (EA) were purchased from Sigma Aldrich. Polyethylene glycol 400 (PEG_400_), citric acid monohydrate 99.5% (CA), propan-2-ol 99% and diphenyl ether 99.5% were obtained from Alfa Aesar.

### 2.2. Synthesis of Magnetic C-Dots

**Scheme 1:**
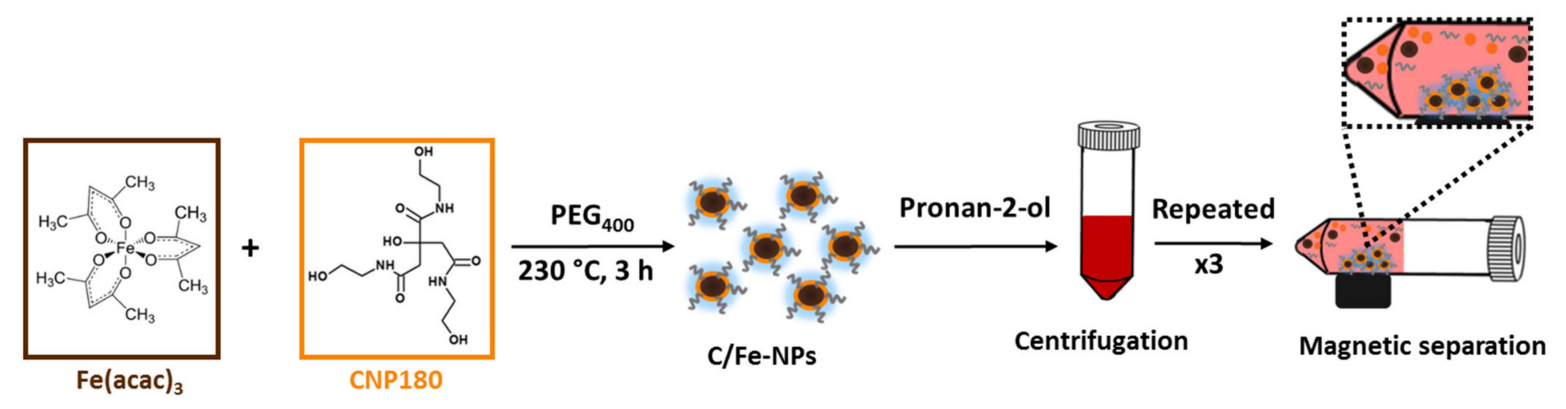
Schematic representation of the process followed for the synthesis of C-Fe/NPs.

Magnetic C-dots were synthesized by thermal decomposition of iron and carbon precursors dispersed in the high boiling point solvent diphenyl ether, as presented in Scheme 1.

A highly photoluminescent C-dots precursor (CNP180) was synthesized via thermal treatment of CA and EA (molar ratio 3:1) at 180 °C, in accordance with a previously reported method [45]. Subsequently, various amounts of CNP180 (0.36 g, 0.45 g, 0.54 g, 0.63 g, 1.08 g) were added into 0.72 g of iron (III) acetylacetonate along with 7.5 mmol of polyethylene glycol 400 and 20 mL of diphenyl ether. Subsequently, the mixture of precursors was heated under reflux with constant stirring for 3 h at 230 °C, to facilitate the formation of an amorphous coating of CNP230 on the iron oxide-based centers. The precipitate was purified by adding 40 mL of propan-2-ol to the mixture, followed by centrifugation at 8000 rpm for 10 min and this step was repeated three times. The C/Fe-NPs thus received were dispersed in distilled water, left for 15 min with a magnet attached to the vial’s wall and the particles not attracted by the magnet were discarded. Finally, all samples were freeze-dried and afterwards stored under ambient conditions.

### 2.3. Characterization

Elemental analysis was conducted with a CHNS Elemental Analyzer (Flash 2000, Thermo Fisher Scientific, Waltham, MA, USA) equipped with a column for oxygen determination that was calibrated against 2,5-(Bis(5-tert-butyl-2-benzo-oxazol-2-yl)thiophene (Thermo Fisher Scientific, Waltham, MA, USA). The carbon, hydrogen, nitrogen analysis was carried out in aluminum pans, while oxygen analysis in silver pans (both types of pans were received from CE Instruments).

Magnetization curves of the solid samples were obtained at temperatures of 296 and 70 K by using a low temperature Faraday balance magnetometer with a continuous flow cryostat. More detailed magnetic measurements were carried out on selected samples at the VSM option of the Quantum Design PPMS apparatus (San Diego, CA, USA) with the oscillation frequency of 40 Hz in the temperature range from 4 to 300 K. The zero field cooled (ZFC) and field cooled (FC) magnetic susceptibility curves were measured in the magnetic field of 100 Oe. The FC data was collected on cooling the nanoparticles in the desired field, whereas the ZFC magnetic susceptibilities were measured after cooling the samples in zero applied field to 4 K, then applying the magnetic field of 100 Oe and recording the data while slowly warming the nanoparticles up to 300 K. Mass magnetization and magnetic susceptibility values were calculated per gram of Fe_3-δ_O_4_ with error bars accounting for uncertainties in the stoichiometry (0 ≤ δ ≤ 0.33) and the measured Fe content.

Transmission electron microscopy (TEM) images were recorded by the FEI Tecnai T12 Spirit TEM (FEI company, Hillsboro, OR, USA) operated at 120 kV. A droplet of 0.05 mg/mL C/Fe-NPs in water was allowed to dry on a carbon coated cupper grid (Agar Scientific, Stansted, UK) and dried under air. The size histogram has been plotted on the basis of 395 fields using a suitable software.

Fourier transform infrared (FTIR) spectra were obtained by Nicolet IS5 spectrometer (Thermo Fisher Scientific, Waltham, MA, USA) within the range of 4000–500 cm^−1^. For each measurement, the samples were scanned 128 times at a resolution of 2 cm^−1^.

Powder X-ray diffraction (PXRD) patterns were recorded by Bruker D2 Phaser diffractometer (Billerica, MA, USA) equipped with a Lynxeye 1-dimensional detector using Cu Kα radiation. Similar volumes of C/Fe-NPs were scanned for 60 min in the 2θ range of 5–80°. Le Bail refinements of the PXRD patterns were carried out with Jana2006 [46]. Bragg peaks were fitted using Lorentzian profiles, and the variation of the half-widths with Bragg angle θ was described by *H*_L_(θ) = *X*/cosθ + ΓL(θ). Here, the first term accounts for isotropic Scherrer broadening due to limited size of diffracting crystallites, while the second term describes instrumental broadening. The latter was assessed from PXRD pattern of high-purity (6N) germanium powder with controlled grain size. Thus, after subtracting the instrumental broadening, the volume averaged size of crystallites was calculated from refined *X* values in degrees using the formula: *D*_V_ = 360 *K*_S_λ/(π2*X*), where λ is the wavelength of the radiation used for the diffraction study, and *K*_S_ denoting the Scherrer constant was assumed to be 1 [47], providing lower bounds for mean sizes of diffracting grains as other contributions to peak broadening (e.g., due to microstrain) were not considered.

Thermogravimetric analysis (TGA) measurements were carried out using a TGAQ500 instrument (TA Instruments, New Castle, UK). Samples were heated over the range of 25–500 °C at a heating rate of 10 °C/min, under continuous nitrogen flow.

Ultraviolet-visible (UV-Vis) spectra of aqueous dispersions of C/Fe-NPs placed in Hellma Analytics quartz cuvette with 1.0 cm pathlength were recorded at room temperature by means of a UV-3600 spectrophotometer (Shimadzu, Torrance, CA, USA).

Photoluminescence (PL) spectra of aqueous dispersions of C/Fe-NPs were collected under excitation wavelengths between 275 and 500 nm using a Horiba Fluoromax spectrofluorometer (Kyoto, Japan) and the samples were placed in Hellma Analytics quartz cuvette with 1.0 cm pathlength.

The quantum yields (QY) of C/Fe-NPs dispersions were determined using anthracene (Sigma Aldrich, Saint Louis, MO, USA) with QY_R_ = 0.27 at λ_ex_ = 365 nm as a standard reference dye and calculated from the equation:

(1) $$\mathrm{QY}={\mathrm{QY}}_{R}\times \left(\frac{I}{I_{R}}\right)\times \left(\frac{A_{R}}{A}\right)\times \left(\frac{\eta^{2}}{\eta_{R}^{2}}\right)$$

where I refers to the integrated fluorescence intensity of the tested material, A refers to its absorbance value and η is the refractive index of the solvent. The subscript R indicates the reference dye. The dye anthracene was dissolved in ethanol (η = 1.36) while the examined samples were dispersed in ultra-pure water (η_R_ = 1.33). To minimize re-absorption effects, absorbance values were kept below 0.10.

Photoluminescence lifetime decays were measured by Edinburgh Instruments LifeSpec-II (Livingston, UK) using two pulsed diode lasers operating at 450 nm (EPL-450) and 375 nm (EPL-375). Time-correlated single photon counting (TCSPC) data acquisition unit processed the detector signals. The PL lifetime decays were recorded with 10,000 peak counts for the aqueous solutions transferred into quartz cuvette (1 cm pathlength) at room temperature over 200 ns time range. The same parameters were kept for recording the instrument response function (IRF), while using dilute aqueous suspension of silica (Ludox HS-30, Sigma Aldrich). The average PL lifetimes (*τ*_avg_) were calculated from the equation:

(2) $$\tau_{\mathrm{avg}}=\frac{\sum \alpha_{i}\tau_{i}^{2}}{\sum \alpha_{i}\tau_{i}}$$

where $\tau_{i}$ is the time component of multiexponential decay fitting and $\alpha_{i}$ is the fractional weight for each time component.

X-ray photoelectron spectroscopy (XPS) was performed using SPECS spectrometer (Berlin, Germany) equipped with Al Kα X-ray radiation source (emitting photons 1486.64 eV) and a PHOIBOS 150 (hemispherical analyzer) with the 20-eV pass energy. The base pressure of the XPS system was 2 × 10^−8^ Pa. CASA XPS software was used to solve the XPS peaks. This was solved by curve fitting with the sum of the lines (Gaussian (70%) and Lorentzian (30%)). Secondary electron background was subtracted using the Shirley procedure. The internal calibration of the XPS spectra was done according to a binding energy value of 284.6 eV for C1s peak.

Cytotoxicity of C/Fe-NPs against epithetical HeLa cervix cancer cell line was evaluated by the standard MTT (3-(4,5-dimethylthiazolyl-2)-2,5-diphenyltetrazolium bromide) assay (Sigma Aldrich). The HeLa cells were cultured in Dulbecco’s modified Eagle medium (DMEM, Thermo Scientific, Waltham, MA, USA) supplemented with 10% fetal bovine serum (Thermo Fisher Scientific) and 1% penicillin-streptomycin (10,000 U/mL, Thermo Fisher Scientific). At first, 50 µL of cell dispersion (1 × 10^5^ cells/mL) were seeded in a 96-well plate and were incubated overnight (37 °C, 5% CO_2_), so that the cells could adhere to the bottom of the well. Subsequently, the cells were incubated with C/Fe-NPs (10–200 µg/mL in DMEM) for 24 h. Then, 20 µL of 5 mg/mL MTT solution was transferred into each well and was further incubated for 2 h. Afterwards, 150 µL of 0.7 M lysis buffer was added into each well to liquefy the formazan crystals. Finally, the optical density (OD) of tested materials was measured by a microplate reader (Thermo Fisher Scientific, Waltham, MA, USA) with a set up wavelength at 595 nm. Cell viability was determined via the relationship:

(3) $$\mathrm{Cell}\;\mathrm{viability}\;(\%)=\frac{{\mathrm{OD}}_{\mathrm{treated}}}{{\mathrm{OD}}_{\mathrm{control}}}\;\times 100$$

where OD_control_ and OD_treated_ were recorded in the absence and the presence of C/Fe-NPs, respectively. Measurements were performed three times and the mean value was calculated.

In vitro fluorescence imaging was done for the HeLa cells cultured in DMEM in the presence of 10% FBS (*v/v*) and 1% penicillin-streptomycin (*v/v*) at 37 °C (5% CO_2_, 95% air) for 24 h. Subsequently, liquid dispersions of 50 µg/mL C/Fe-NPs were added into each well with the HeLa cells and incubated for a further 24 h. Afterwards, the media was aspirated and the cells were washed three times using PBS buffer (pH 7.2). The fluorescence imaging in live cells was performed with the use of Zeiss Axio Scope A1 microscope (Oberkochen, Germany), under a bright field, and under excitation wavelengths of 366, 488 and 512 nm.

Antimicrobial studies—the culturing protocol has been reported previously [48]. Briefly, an inoculated nutrient broth with a single loop of either *Escherichia coli* (*E. coli*) or *Staphylococcus aureus* (*S. aureus*) was incubated for 24 h at 37 °C, while using SciQuip Incu-Shake MIDI orbital shaker (SciQuip Ltd., Newtown, Wem, Shropshire, UK) set as 200 rpm. Afterwards, the bacteria cultures were centrifuged twice, followed by the disposal of supernatant. Then, the cultures were resuspended and further diluted in broth, in order to obtain absorbance values close to a 0.5 McFarlane standard.

The method used for antimicrobial testing required dispersion of 0.1 g of C/Fe-NPs in 1 mL nutrient broth, which had to be further autoclaved. Afterwards, 1 mL of tested bacterial strain and 8 mL sterile nutrient broth were added into each dispersion and the mixture was incubated for 24 h at 37 °C under 200 rpm stirring. Subsequently, 1 mL aliquot was taken from each suspension and further serially diluted by transferring 100 µL of it into 900 µL Ringer’s solution with ¼ strength. The dilution process was repeated until the number of bacterial colonies fell to between 30 and 300. Subsequently, 100 µL of each one from the series of diluted suspension was spread onto agar-gelled nutrient plates, with the use of sterile plastic spreader. In the final step, the agar plates were incubated at 37 °C for 24 h and the number of bacterial colonies was estimated. The comparison with the control plate allowed calculation of the overall percentage of bacterial colonies decrease accordingly to the following equation:

(4) $$\mathrm{Bacterial}\;\mathrm{colony}\;\mathrm{decrease}\;(\%)=\frac{\mathrm{Control}\;-\;\mathrm{Test}}{\mathrm{Control}}\;\times 100$$

Measurements for each of the C/Fe-NPs were performed three times and their mean values are reported.

## 3. Results and Discussion

**Figure 1:**
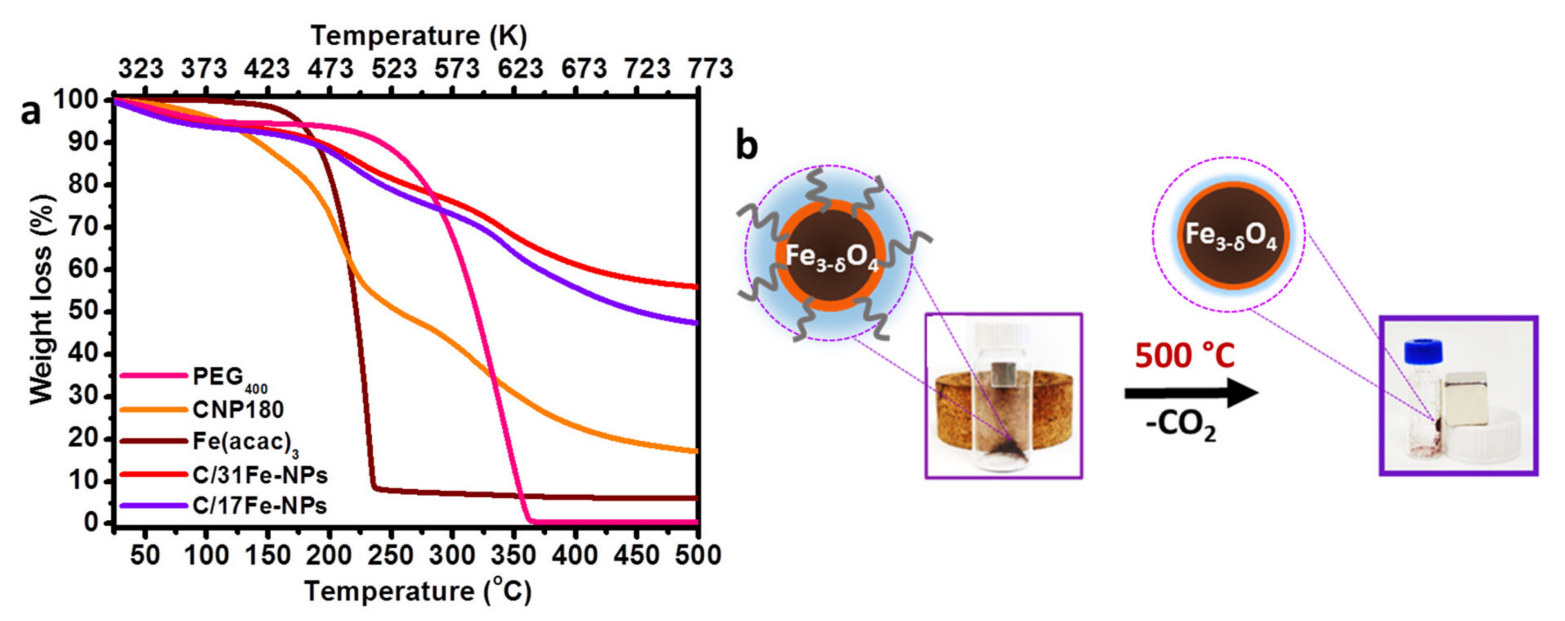
(**a**) TGA curves of C/17Fe NPs, C/31Fe NPs, PEG_400_, CNP180 and Fe(acac)_3_; (**b**) photos demonstrating the magnetic nature of C/17Fe NPs prior (left image) and after (right image) the TGA scan presented in (**a**).

It has been previously demonstrated that pyrolysis at 230 °C of the fluorescent precursor CNP180 (derived via thermal treatment of EA and CA at 180 °C for 30 min as discussed in the experimental section) leads to the formation of well-defined spherical NPs (referred hereafter as CNP230) with average size of 19 nm [45]. In this study CNP180 was subjected to pyrolysis at 230 °C, but in the presence of varying amounts of Fe(acac)_3_ and upon the addition of PEG_400_ in order to synthesize a series of well-defined C/Fe-NPs. Preliminary work indicated that both the PL intensity (Supplementary Materials, Figure S1) and the crystallinity (Supplementary Materials, Figure S2) of the C/Fe-NPs are optimized following 3 h of pyrolytic treatment, thus this pyrolysis time was kept constant for all experiments described here. TGA analysis suggests that Fe(acac)_3_ abruptly decomposes at temperatures around 230 °C (loss weight 91% at 236 °C), while CNP180 and PEG_400_ are much less prone to degradation in this temperature region (Figure 1a).

**Scheme 2:**
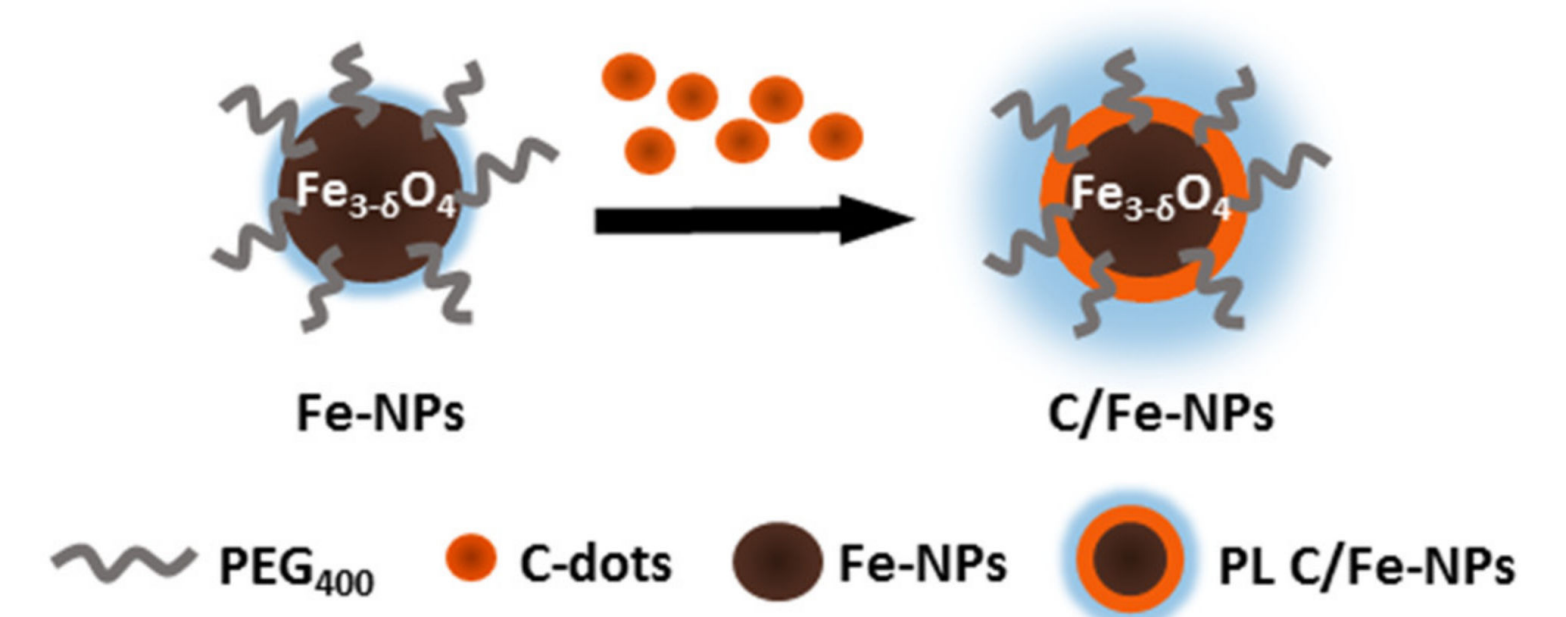
Scheme presenting the build-up of C/Fe-NPs.

The TGA profiles point to the early formation of iron-rich nuclei and the subsequent formation of carbon-rich corona (Scheme 2). Interestingly, C/17Fe-NPs continue being attracted by the magnet even after the completion of the TGA scan (up to 500 °C) (Figure 1b).

On the basis of this approach and by varying the weight ratio between Fe(acac)_3_ and CNP180, a series of C/Fe-NPs was synthesized. Elemental analysis (Table 1) suggested that the iron content of the C/Fe-NPs varies between 17 and 31 wt%; hereafter the notation C/17Fe-NPs is used to indicate 17 wt% iron content, C/31Fe-NPs to indicate 31 wt% iron content, etc. At the same time, the carbon content monotonically decreases from 36.3 for C/17Fe-NPs to 26.9 wt% for C/31Fe-NPs. Within this family of materials, the oxygen, nitrogen and hydrogen contents are between 34.7 and 36.2 wt%, 3.9 and 6.5 wt% and 3.6 and 4.3 wt%, respectively.

A major advantage of our synthetic strategy is a facile one-pot and single-step fabrication procedure, which has potential to be easily scaled up and modified. In contrast, previous reports refer to tedious multistep synthetic procedures [16,29,36,38,39,40,41,42,43,44].

**Figure 2:**
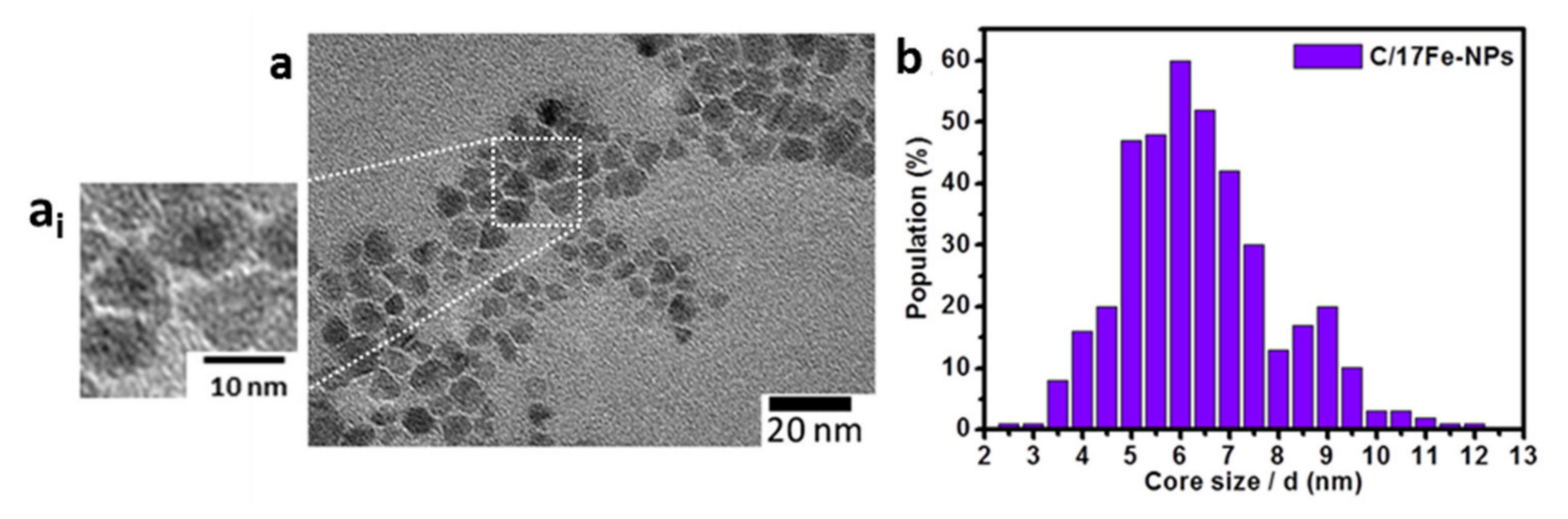
(**a**) TEM image with (a_i_) an inset demonstrating nucleus-corona structure and (**b**) size histogram of C/17Fe-NPs plotted on the basis of 395 fields.

The TEM image of C/17Fe-NPs (Figure 2a) reveals the presence of nanoparticles with center-coating structure. In addition, C/17Fe-NPs demonstrate a relatively high level of homogeneity with an average particle size 6.4 nm (Figure 2b). The good separation between particles might be attributed to the presence of the carbogenic corona and the passivating role of PEG_400_ layer.

**Figure 3:**
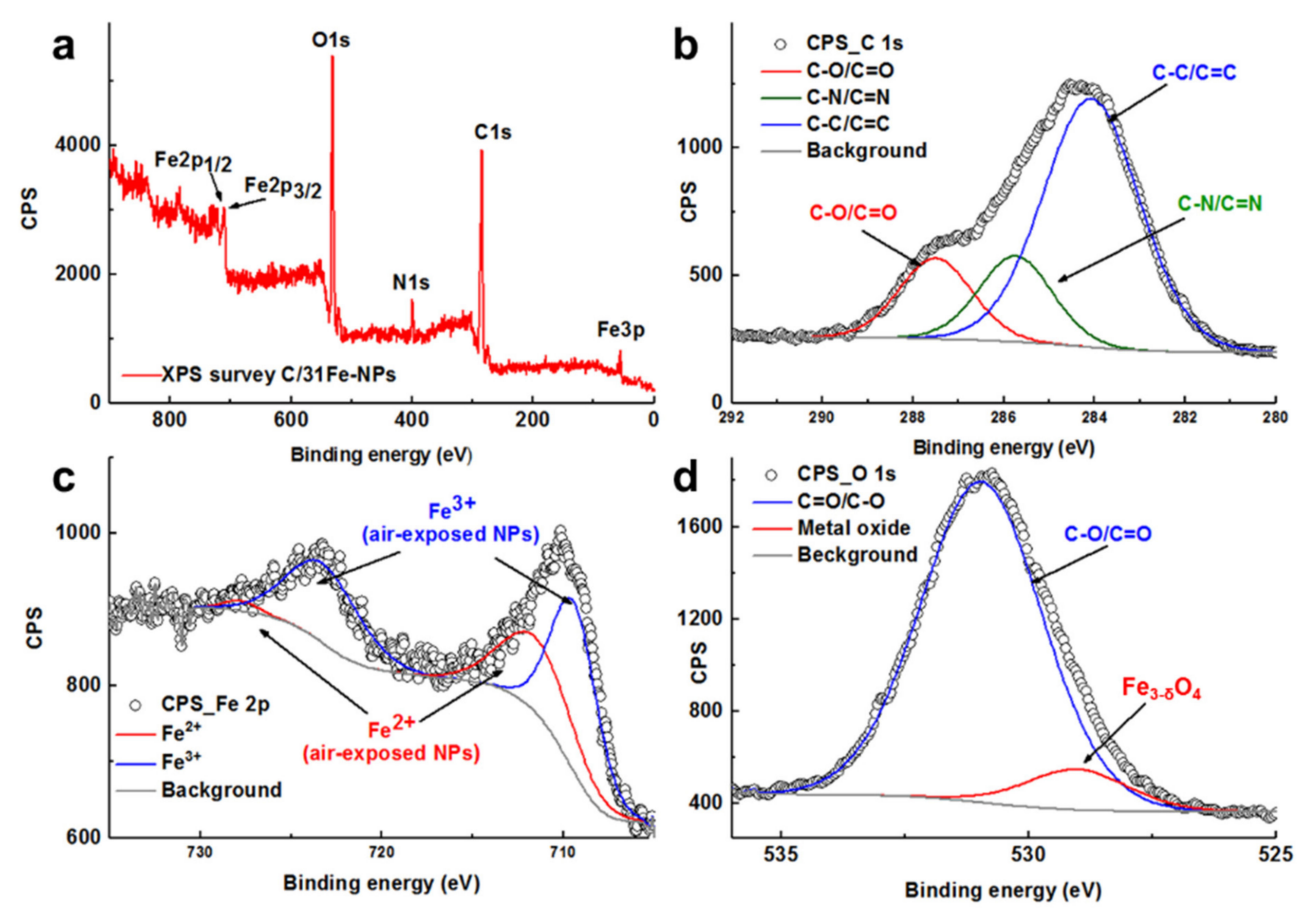
(**a**) The XPS survey together with deconvoluted (**b**) C1s, (**c**) Fe2p and (**d**) O1s XPS spectra of C/31Fe-NPs. Circular points refer to the data collected, while the solid lines refer to the fitted curves.

The XPS survey spectrum of C/31Fe-NPs (Figure 3a) indicates a very low iron content on the surface, thus confirming that iron is predominantly located at the center. The high-resolution C1s spectra of C/31Fe-NPs (Figure 3b) can be deconvoluted into three peaks located at 284.1 eV, 285.7 eV and 287.5 eV that correspond to C-C/C=C, C-N/C=N and C-O/C=O, respectively [34,38,49]. The high-resolution Fe2p spectra of C/31Fe-NPs (Figure 3c) demonstrated the occurrence of Fe2p_3/2_ and Fe2p_1/2_ peaks centered around 722 eV and 711 eV, which point to Fe^3+^ and Fe^2+^ oxidation states of the air exposed C/Fe-NPs (assuming a certain level of porosity for the carbogenic corona), respectively [35,38,49,50]. The high-resolution O1s spectra of C/31Fe-NPs (Figure 3d) can be deconvoluted into two peaks located around 531 eV and 529 eV, which further confirm the presence of carboxyl groups and iron metal oxides, respectively. Similar results were obtained for other C/Fe-NPs.

**Figure 4:**
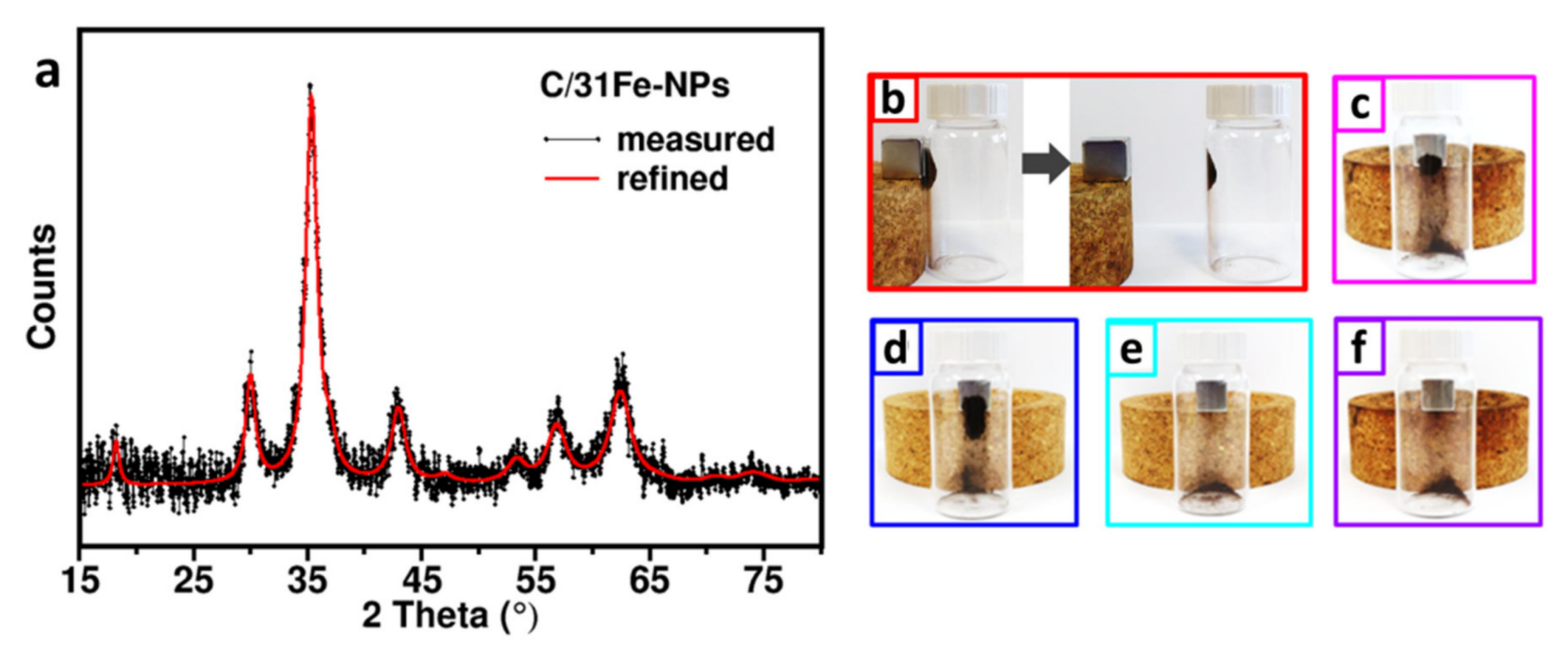
(**a**) PXRD pattern of C/31Fe-NPs (black points refer to experimental data and the red line refer to the refined profiles). The photos of (**b**) C/31Fe-NPs, (**c**) C/27Fe-NPs, (**d**) C/24Fe-NPs, (**e**) C/23Fe-NPs, (**f**) C/17Fe-NPs (all enclosed in glass vials) in the presence of an external magnet.

PXRD patterns for C/31Fe-NPs (Figure 4a), C/27Fe-NPs and C/24Fe-NPs (Supplementary Materials, Figure S3a) match well with the PDF card Nos. 19-0629 and 00-004-0755 of spinel-type (magnetite/maghemite) phase. The Bragg peaks appear much weaker for C/23Fe-NPs and C/17Fe-NPs (Supplementary Materials, Figure S3b), as expected due to their lower iron content. Profile refinements of the PXRD patterns (example in Figure 4a) indicated that the spinel-type phase is nanocrystalline, with the grain size decreasing slightly as the fraction of the iron oxides is reduced (Table 2).

As shown in Figure 4b–f, only C/31Fe-NPs, C/27Fe-NPs and C/24Fe-NPs are strongly attracted by a magnet, while C/23Fe-NPs and C/17Fe-NPs fail to do so.

**Figure 5:**
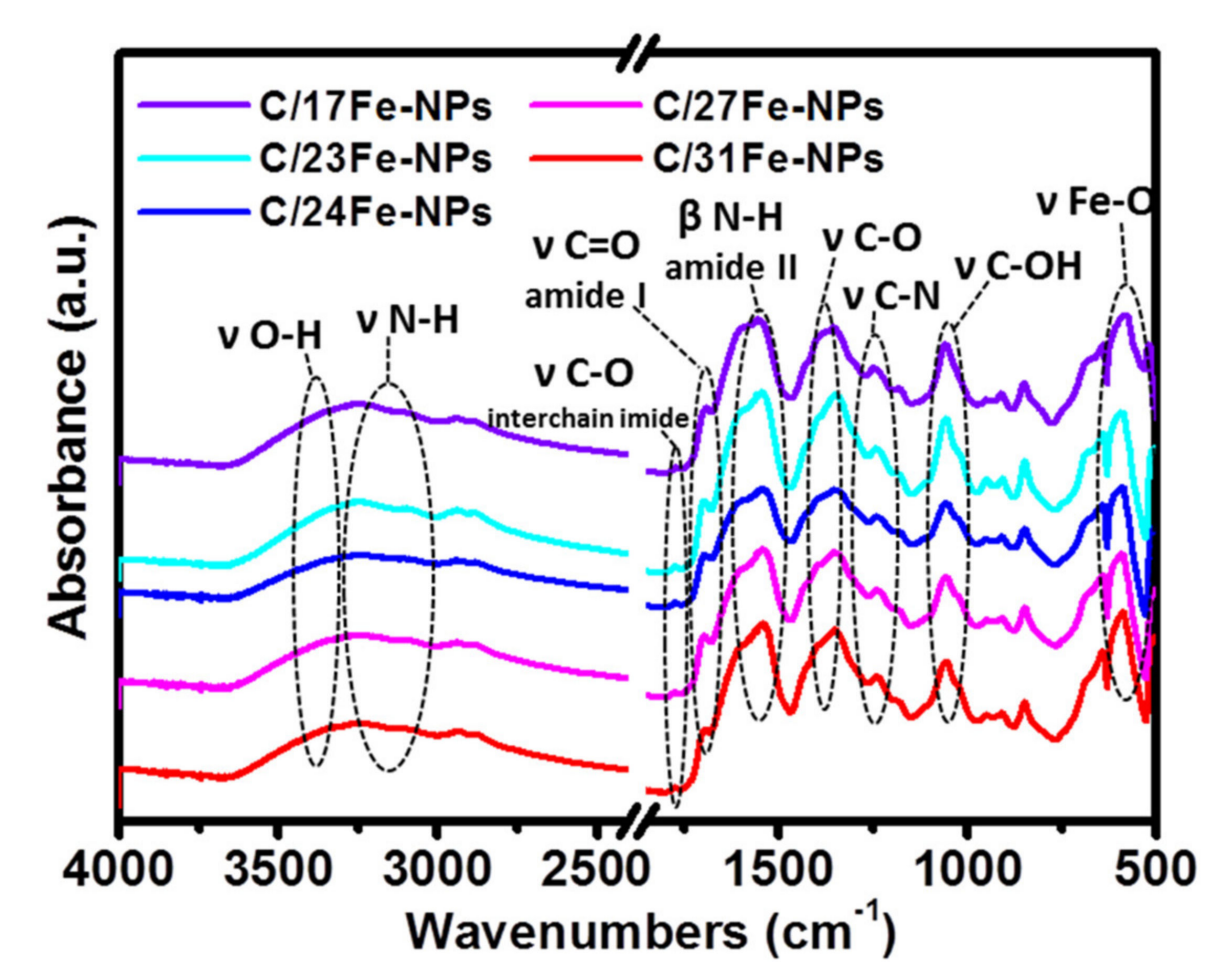
The FTIR spectra of C/Fe-NPs.

The FTIR spectra of C/Fe-NPs (Figure 5) show the presence of peaks centered around 588 cm^−1^ (Fe-O stretching vibration), 1054 cm^−1^ (C-OH stretching vibration), 1232–1352 cm^−1^ (C-O and C-N stretching vibrations), 1750 cm^−1^ (C=O stretching vibration), 2900 cm^−1^ (C-H symmetric and asymmetric stretching vibrations) and 3240 cm^−1^ (N-H and O-H stretching vibrations) [16,34,38,39,49,51]. The peaks in the range of 1540–1697 cm^−1^ are associated with the C=O stretching and N-H in-plane bending vibrations of amide I and II absorption band, respectively [35,39,40,49,52]. The FTIR spectroscopy confirms the presence of oxygen-rich groups on the C/Fe-NPs surface that render the particles dispersible in polar media.

**Figure 6:**
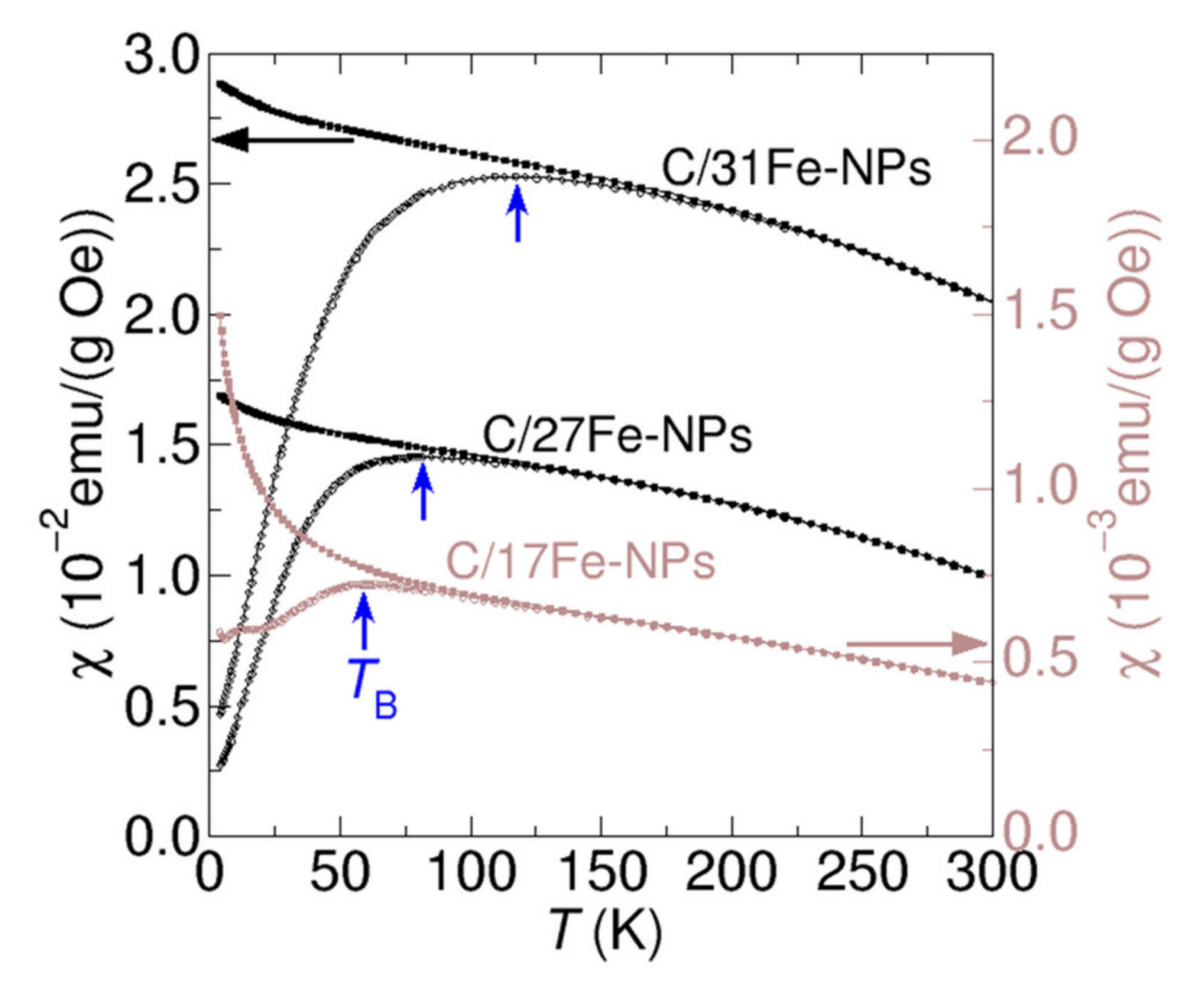
Temperature dependencies of magnetic susceptibilities for selected C/Fe-NPs measured in the ZFC (empty dots) and FC (filled dots) modes using the applied magnetic field of 100 Oe. Data shown in black was plotted on the left vertical axis, whereas brown data points are using the brown vertical axis located on the right-hand side. The blocking temperatures are indicated by vertical blue arrows.

Figure 6 shows typical ZFC and FC curves recorded from selected C/Fe-NP samples in magnetic field of 100 Oe. The magnetic susceptibilities obtained in the ZFC mode show broad peaks signifying a transition from the low temperature blocked state to the superparamagnetic regime at high temperatures [53]. The blocking temperatures (*T*_B_) corresponding to the maxima in the ZFC curves are higher for samples with larger Fe content, in agreement with the conjecture that those samples contain bigger Fe-NPs which are expected to become superparamagnetic at higher temperatures [54].

**Figure 7:**
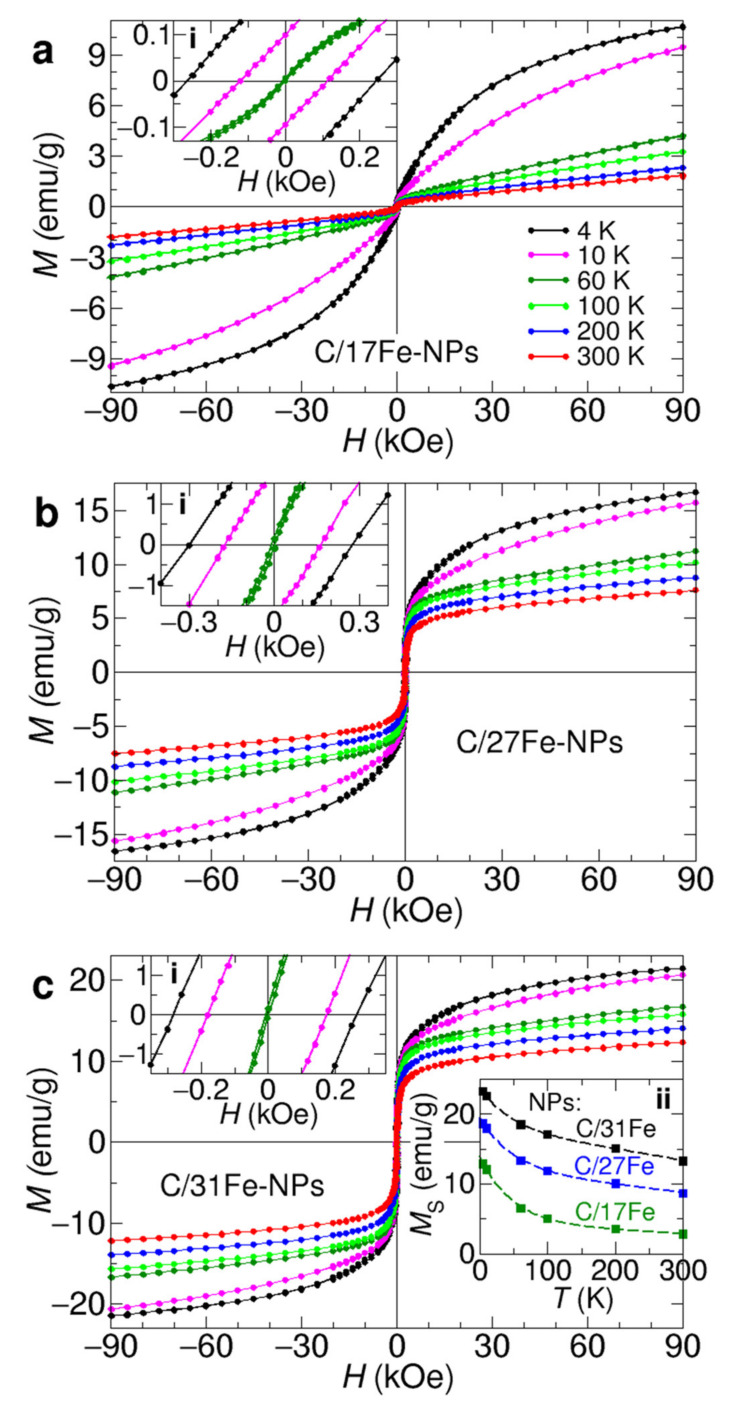
Magnetic hysteresis loops recorded at temperatures of 4 K (black dots), 10 K (pink dots), 60 K (dark greed dots), 100 K (green dots), 200 K (blue dots) and 300 K (red dots) from C/17Fe-NPs (**a**), C/27Fe-NPs (**b**) and C/31Fe-NPs (**c**). Insets i show expanded *M*-*H* plots near the origin. Inset ii of (**c**) presents the saturation magnetization as a function of temperature for C/17Fe-NPs (black squares), C/27Fe-NPs (blue squares) and C/31Fe-NPs (dark green squares), together with fits using Equation (5) (dashed lines).

The isothermal magnetization measurements shown in Figure 7 further indicate that C/Fe-NPs exhibit a predominant superparamagnetic behavior close to room temperature. Upon cooling, onset of hysteresis loops are observed in the *M*(*H*) curves at temperatures between 150 and 50 K indicating transitions to the blocked state. As shown in insets i of Figure 7, the coercivity increases with decreasing temperature, reaching values of 0.25–0.30 kOe at 4 K.

The magnetization of C/Fe-NPs does not reach saturation even at 90 kOe and 4 K. Therefore, the saturation magnetization (*M*_s_) was estimated by extrapolating the magnetization versus 1/*H* to the limit when the inverse magnetic field approaches zero. The resulting *M*_S_ values for selected C/Fe-NPs are shown in inset ii of Figure 7c. When calculated per gram of Fe_3-δ_O_4_ (0 ≤ δ ≤ 0.33), the *M*_s_ values at 4 K fall within 50–56 emu/g-Fe_3-δ_O_4_ and are smaller than 90 and 84 emu/g expected for bulk magnetite and maghemite, respectively [55]. We note that substantially reduced *M*_s_ is characteristic for Fe-NPs with sizes in the surface of spinel cores [53,56,57,58,59,60,61,62,63]. The saturation magnetization of C/Fe-NPs decreases the range of a few nanometers and was attributed to the presence of disordered spins on upon heating, signifying thawing of the surface spins [64,65,66].

As illustrated in inset ii of Figure 7c, the temperature dependencies of *M*s can be well described by the phenomenological formula [66,67].

*M*_S_(*T*)/*M*_S_(0) = (1 − *B T*^3/2^) + *A* exp(−*T*/*T*_f_) (5)

in which the first term accounts for a Bloch-like variation of magnetization expected for magnetically ordered core moments whereas the second term represents the effect of freezing of disordered surface spins in a spin-glass-like state at temperatures below *T*_f_. Fitting Equation (5) to the *M*s(*T*) data resulted in parameters collated in Table 3. The obtained values of the Bloch constant *B* are similar to those previously reported [67,68] for NPs of Fe_3_O_4_ and γ-Fe_2_O_3_ with comparable sizes. The *A* constant scales with the relative amount of surface spins compared to magnetically ordered core moments. Therefore, the observed systematic increase in the *A* values as the Fe content is reduced indicates a growing fraction of surface spins, in line with an increasing surface-to-volume ratio expected when spinel NPs are getting smaller. Simultaneously, the saturation magnetization ascribed to the magnetically ordered spinel cores dwindles, providing a further indication for a decrease in sizes of Fe-centers as the Fe content is reduced.

**Figure 8:**
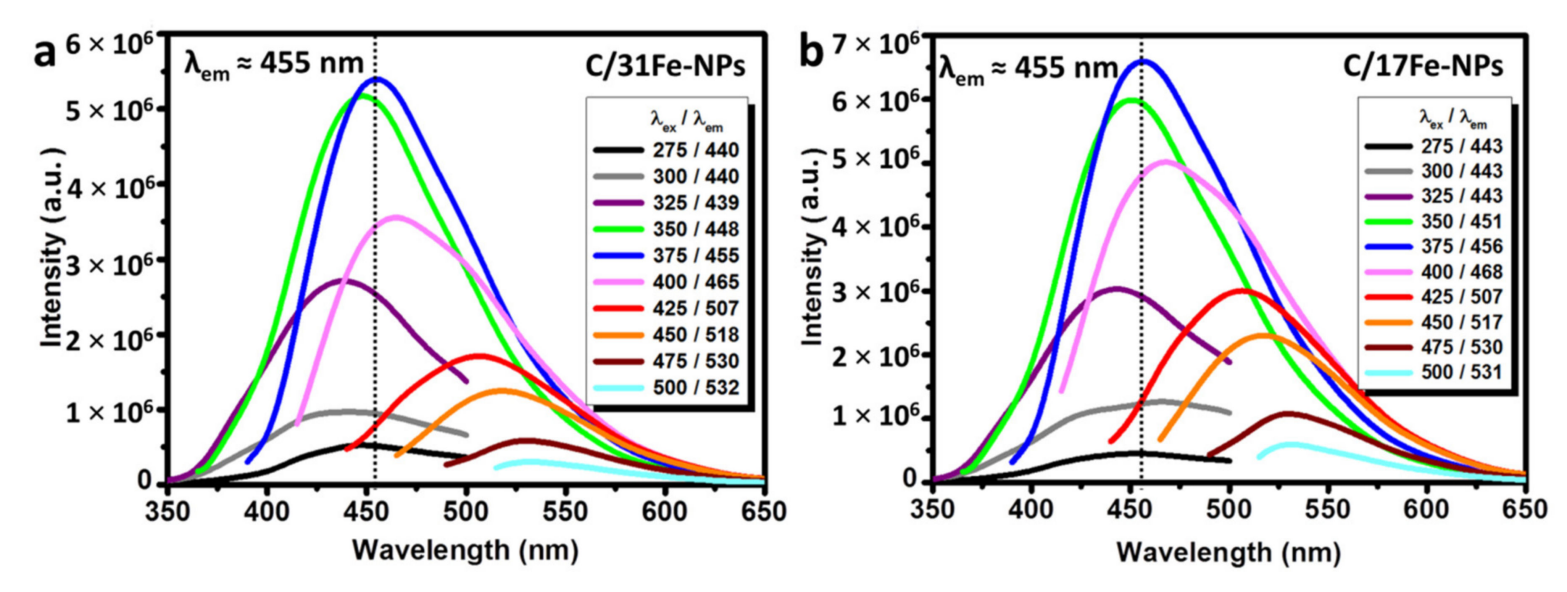
PL spectra of aqueous dispersions of (**a**) C/31Fe-NPs and (**b**) C/17Fe-NPs.

The UV-Vis spectra of magnetic C-dots (Supplementary Materials, Figure S4a) reveals the presence of absorption peaks at 270 nm and a hump around 320 nm, which are attributed to π-π* electron transitions of polyaromatic chromophores as well as n-π* electron transitions of carbonyl (C=O) and amine (C-N) functional groups [33,35,39,44]. Although the absorptivity of aqueous dispersions of C/Fe-NPs are rather low [39,49,69,70], their aqueous dispersions display a strong blue luminescence under UV light (Supplementary Materials, Figure S4b). The fluorescence spectra of C/31Fe-NPs and C/17Fe-NPs (Figure 8) show an excitation wavelength independent contribution (λ_em_ = 455 nm) that has been attributed to the presence of citrazinic acid and an excitation wavelength dependent emission at higher λ_ex_ that is characteristic for carbogenic NPs [45]. This fluorescent mode arises from surface emission states, edge defects, self-trapped excitons, crosslink enhanced emission and quantum conferment in the context of sp^2^ islands within ab sp^3^ matrix [16,34,35,39,50]. Typically C-dots exhibit the strongest PL within the blue region [71], but red-shifted emission is achieved via π-conjugation of nano-domains, pronounced surface oxidation and incorporation of heteroatoms, such as N, S and P [72].

As shown in Supplementary Materials, Figure S5, the QY at λ_ex_ = 365 nm increases with carbon content from 6% for C/31Fe-NPs, C/27Fe-NPs and C/24Fe-NPs to 7% for C/23Fe-NPs and 9% for C/17Fe-NPs. These values are consistent with previous studies that reported QY ranging from 4.6 to 8% for related systems [33,49,50]. The time-resolved PL lifetime decays at λ_ex_ = 375 nm (Supplementary Materials, Figure S6) and 450 nm (Supplementary Materials, Figure S7) of aqueous dispersions of C/Fe-NPs follow complex multi-exponential decay profiles, in agreement with previous studies [39]. The average PL lifetimes (*τ*_avg_) of C/Fe-NPs range from 12.43 up to 12.80 ns (Supplementary Materials, Figure S6) compared to *τ*_avg_ = 13.06 ns for CNP230 (Supplementary Materials, Figure S8), *τ*_avg_ = 1.5–4 ns for the auto fluorescence of the cells, *τ*_avg_ = 1–5 ns for organic dyes and *τ*_avg_ = 6.4 ns for citrazinic acid. These data indicate a weak dependence on the iron content and a rather limited effect of the magnetic core on the microenvironment of the excited states within the carbogenic corona. The larger *τ*_avg_ observed for C/Fe-NPs carry promise for cellular labelling, tissue analysis and microfluidic devices [73].

**Figure 9:**
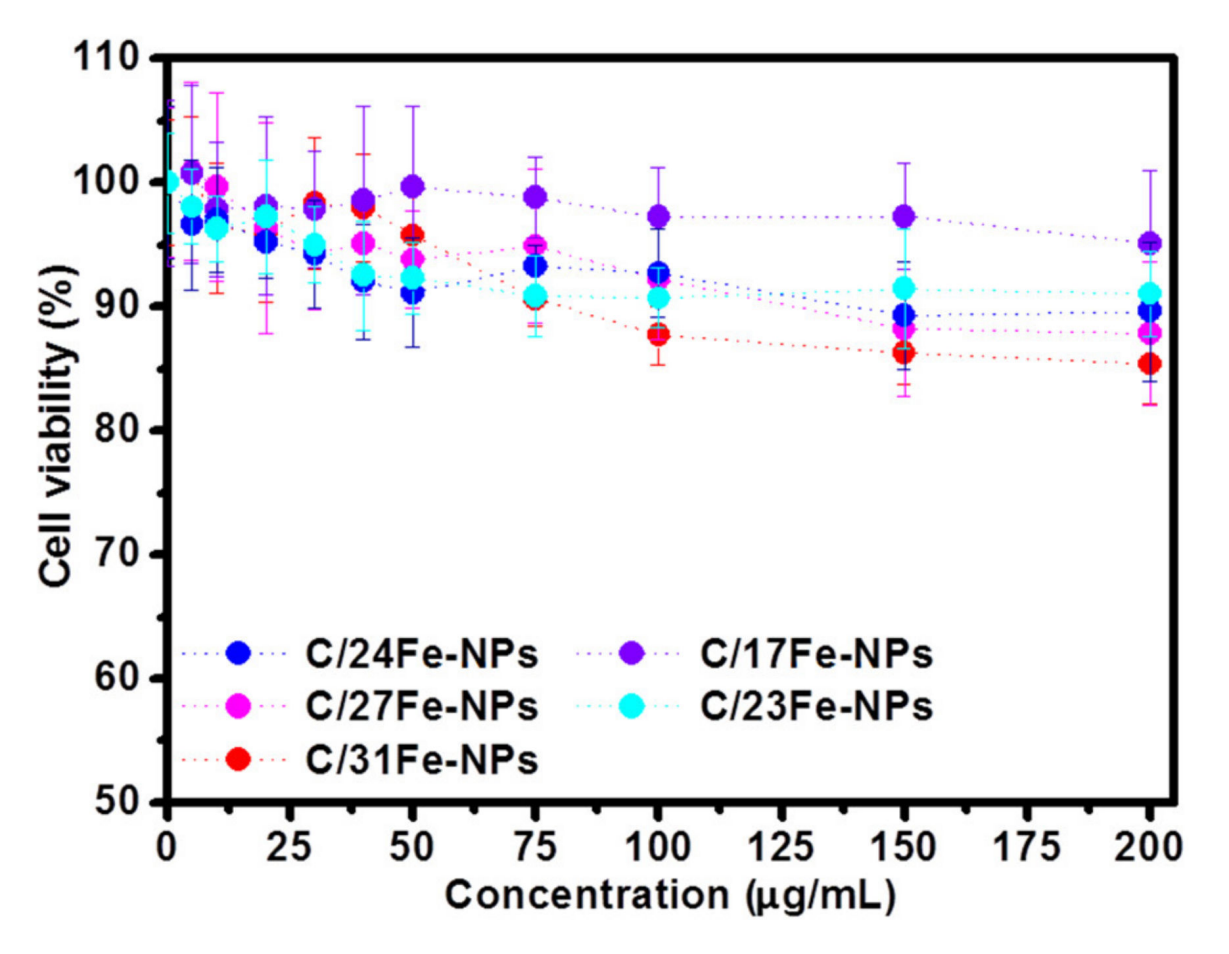
The viability of HeLa cells derived from MTT assay following 24 h incubation with C/Fe-NPs.

The viability of HeLa cancer cells incubated with various concentrations of C/Fe-NPs (as estimated using standard MTT assays) is shown in Figure 9. At the highest concentration studied (200 µg/mL), the viability of HeLa cells was found to be 95% for C/17Fe-NPs, 91% for C/23Fe-NPs, 90% for C/24Fe-NPs, 87% for C/28Fe-NPs and 86% for C/31Fe-NPs. For comparison, the viability of Hela cells incubated with 200 µg/mL of CNP230 (Supplementary Materials, Figure S9) and Fe-NPs is 97 and 61%, respectively. In principle, the cytotoxicity of NPs critically depends upon the nature of the surface functional groups [74]. Previous studies demonstrated the low toxicity of C-dots derived from CA and EA [39], in contrast to the toxic nature of heavy-metal based quantum [75] and the cell type-specific activity of the Fe-NPs [76,77].

**Figure 10:**
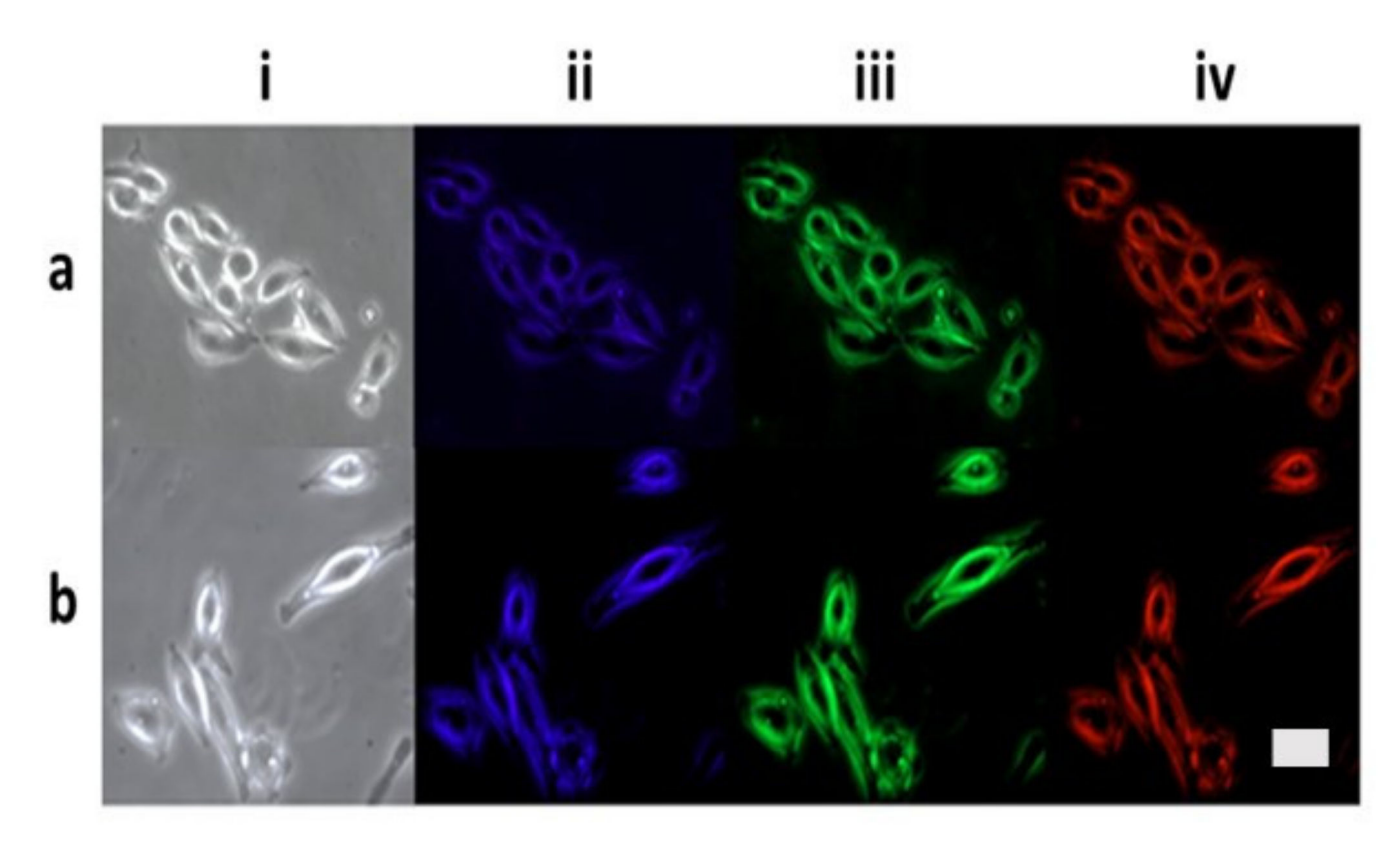
Fluorescent microscope images of HeLa cells following incubation with: (**a**) C/31Fe-NPs and (**b**) C/17Fe-NPs under the bright field (i) as well as the UV (ii), blue (iii) and green (iv) excitation wavelengths. The scale bar corresponds to 100 µm.

Fluorescent microscope images of HeLa cells following incubation with C/31Fe-NPs (Figure 10a) and C/17Fe-NPs (Figure 10b) for 24 h indicated that C/Fe-NPs penetrate the cells and are predominantly accumulated in the cytoplasm [40]. When illuminated with blue, green and red radiation, the internalized C/Fe-NPs can reveal morphological information regarding both the cytoplasm and the membranes.

Overall C/Fe-NPs with low cytotoxicity and superior fluorescent properties act as efficient surveillance probes, while having the added advantage of being able to be directed via an external magnetic field to targeted destinations [35].

Finally, the antimicrobial activities of CNP230 and C/Fe-NPs were tested against *S. aureus* and *E. coli* (a representative Gram-positive and Gram-negative strain, respectively). As summarized in Table 4, C/Fe-NPs demonstrate excellent antimicrobial activity after incubation with bacterial species for 24 h at 37 °C, causing 99.0–99.9% and 99.5–99.9% reduction in *E. coli* and *S. aureus* colonies, respectively. Very similar performance was observed for CNP230 as well as for a variety of C-dots-based materials [78], while Fe-NPs synthesized as a control sample via the same synthetic approach but without the carbogenic coating displayed 91.4% and 99.9% decrease in *E. coli* and *S. aureus* under identical conditions, respectively [37,79]. The photo-activated antimicrobial mechanism of C-dots has been largely associated with the formation of reactive oxygen species (ROS), while the occurrence of nanoparticle-bacterial membrane electrostatic attractions can damage the membrane leading to leakage of the cytoplasm [80]. The data suggest that C/Fe-NPs could be viewed as highly reliable antimicrobial agents, able to combat bacterial contamination, preventing infectious diseases [81]. We note that a magnet can be employed to direct (and eventually withdraw) the C/Fe-NPs in otherwise inaccessible locations, affording disinfection and bacterial decontamination in a controlled manner.

## 4. Conclusions

**Figure 11:**
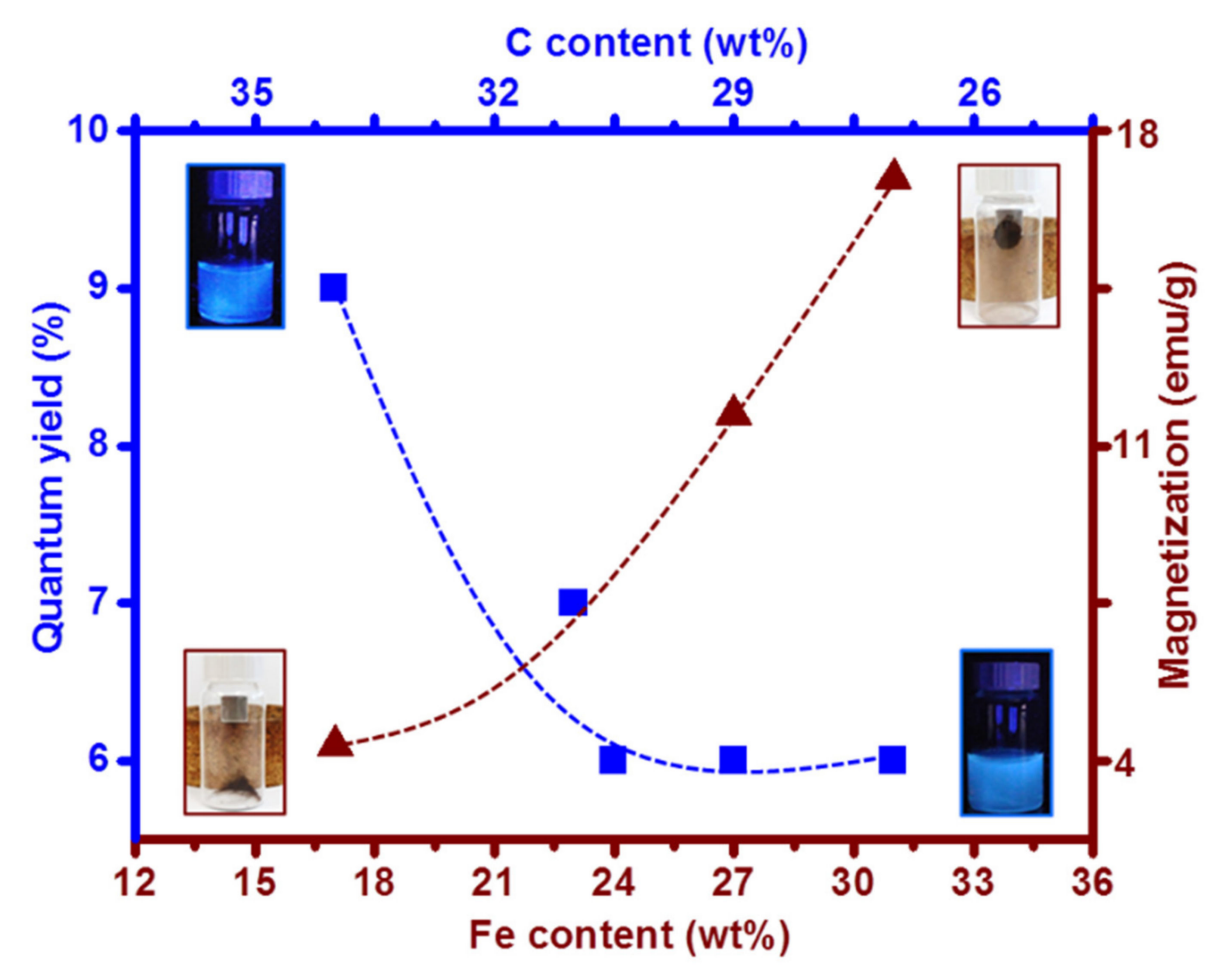
Quantum yield and magnetization of a series of C/Fe-NPs with varying C and Fe contents. Blue squares correspond to QY as a function of C content and brown triangles correspond to magnetization as a function of Fe content.

The study presents a facile strategy to fine-tune the structure and the chemical composition and, thereby, the optical and magnetic properties of C/Fe-NPs. A systematic investigation within this series of materials indicated that the C/17Fe-NPs with carbon content 36 wt% and iron content 17 wt% show QY close to 9% and magnetization 4.1 emu/g, while C/31Fe-NPs with carbon content 27 wt% and iron content 31 wt% show QY 6% and magnetization 17.7 emu/g (Figure 11). C/Fe-NPs are non-toxic materials that can be used as advanced diagnostic tools, while showing excellent antimicrobial activity against *E. coli* and *S. aureus*.

## Figures and Tables

**Table 1:** Elemental analysis of C/Fe-NPs.

	C (wt%)	H (wt%)	N (wt%)	O (wt%)	Fe (wt%)
CNP230	43.30	5.39	9.03	42.28	-
C/17Fe-NPs	36.30	4.66	6.50	35.16	17.38
C/23Fe-NPs	31.45	4.29	5.38	36.11	22.77
C/24Fe-NPs	31.03	4.16	4.86	36.16	23.79
C/27Fe-NPs	28.90	3.97	4.33	35.87	26.93
C/31Fe-NPs	26.85	3.62	3.85	34.68	31.00

**Table 2:** Volume weighted diameters of nanoparticle cores D_V_ estimated from powder X-ray diffraction patterns as detailed in the Materials and Methods Section.

Sample	D_V_ (nm)
C/31Fe-NPs	4.6 ± 0.6
C/27Fe-NPs	4.0 ± 0.7
C/24Fe-NPs	3.5 ± 0.8
C/23Fe-NPs	3.0 ± 0.8
C/17Fe-NPs	2.7 ± 0.9

**Table 3:** Magnetic parameters derived from least-squares fits of the *M*_S_(*T*) data shown in the inset ii of Figure 7c with Equation (5).

Sample	A (−)	B (10^−5^ K^−3/2^)	T_f_ (K)	M_s_(0) (emu/g)
C/31Fe-NPs	0.42 ± 0.05	4.0 ± 0.2	52 ± 2	17.7 ± 0.5
C/27Fe-NPs	0.75 ± 0.08	4.2 ± 0.2	53 ± 2	11.7 ± 0.4
C/17Fe-NPs	2.57 ± 0.15	5.2 ± 0.3	49 ± 2	4.1 ± 0.1

**Table 4:** Antimicrobial performance of CNP230 and C/Fe-NPs.

**Material**	*Escherichia coli*	*Staphylococcus aureus*
	**% decrease**	**% decrease**
	**24 h**	**24 h**
CNP230	99.9	99.9
C/17Fe-NPs	99.9	99.9
C/23Fe-NPs	99.9	99.9
C/24Fe-NPs	99.9	99.9
C/27Fe-NPs	99.0	99.5
C/31Fe-NPs	99.5	99.9

## Data Availability

The data presented in this study are available on request from the corresponding author.

## Supplementary Materials

The following are available online at https://www.mdpi.com/article/10.3390/nano12040674/s1, Figure S1. (a) The PL spectra (λ_ex_ = 375 nm) of aqueous dispersions of C/Fe-NPs prepared from identical reactant mixtures with C/31Fe-NPs, but at various times of pyrolysis (b); Figure S2. PXRD patterns of C/Fe-NPs prepared from identical reactant mixtures with C/31Fe-NPs but at various times of pyrolysis; Figure S3. (a) PXRD patterns of C/27Fe-NPs (magenta) and C/24Fe-NPs (blue) along with (b) PXRD patterns of C/23Fe-NPs (cyan) and C/17Fe-NPs (violet); Figure S4. (a) Absorption spectra of aqueous dispersions of 0.01 mg/mL C/Fe-NPs; (b) Photos of C/31Fe-NPs (red), C/24Fe-NPs (blue) and C/17Fe-NPs (purple) under daylight and ultraviolet light; Figure S5. Integrated PL intensity of (a) C/17Fe-NPs, (b) C/23Fe-NPs, (c) C/24Fe-NPs, (d) C/27Fe-NPs and (e) C/31Fe-NPs in water as a function of optical absorbance at 365 nm; Figure S6. Time-resolved fluorescence decay profiles for aqueous solutions of (a) C/17Fe-NPs, (b) C/23Fe-NPs, (c) C/24Fe-NPs, (d) C/27Fe-NPs, (e) C/31Fe-NPs (e) at λ_ex_= 375nm; Figure S7. Time-resolved fluorescence decay profiles for aqueous solutions of (a) C/17Fe-NPs, (b) C/23Fe-NPs, (c) C/24Fe-NPs, (d) C/27Fe-NPs, (e) C/31Fe-NPs at λ_ex_= 450 nm; Figure S8. Time-resolved fluorescence decay profiles for aqueous solutions of CNP230 recorded at λ_ex_= 375 nm (grey colour) and λ_ex_= 450 nm (dark yellow colour); Figure S9. (a) The MTT assay results for HeLa cells incubated with CNP230 for 24 h; (b) The fluorescence microscope images of HeLa cells with internalized CNP230.
