# Supplementary material for: Carbon Dots/Iron Oxide Nanoparticles with Tuneable Composition and Properties
Source: Nanomaterials (Basel). 2022 Feb 17;12(4):674. doi: 10.3390/nano12040674 (PMC8875257; doi:10.3390/nano12040674)
Supplement: Supplementary file 1 [file nanomaterials-12-00674-s001.zip › nanomaterials-1566340-supplementary.pdf]

nanomaterials

MDPI

Article

Carbon Dots/Iron Oxide Nanoparticles with Tuneable Composition and Properties

Joanna D. Stachowska [1], Monika B. Gamża [2,3], Claire Mellor [4], Ella N. Gibbons [1], Marta J. Krysmann [1], Antonios Kelarakis [3,*], Elżbieta Gumieniczek-Chłopek [5], Tomasz Strączek [5], Czesław Kapusta [5] and Anna Szwajca [6]

1 School of Dentistry, University of Central Lancashire, Preston PR12HE, UK; jstachowska@uclan.ac.uk (J.D.S.); engibbons3@uclan.ac.uk (E.N.G.); mkrysmann@uclan.ac.uk (M.J.K.)
2 Jeremiah Horrocks Institute for Mathematics, Physics, and Astrophysics, University of Central Lancashire, Preston PR12HE, UK; mgamza@uclan.ac.uk
3 UCLan Research Centre for Smart Materials, School of Natural Sciences, University of Central Lancashire, Preston PR12HE, UK
4 School of Phycology and Computer Science, University of Central Lancashire, Preston PR12HE, UK; cmellor3@uclan.ac.uk
5 Faculty of Physics and Applied Computer Science, AGH University of Science and Technology, Mickiewicza Ave. 30, 30-059 Krakow, Poland; echlopek@agh.edu.pl (E.G.-C.); tomasz.straczek@fis.agh.edu.pl (T.S.); kapusta@agh.edu.pl (C.K.)
6 Faculty of Chemistry, Adam Mickiewicz University, Umultowska 89b, 61-614 Poznań, Poland; anna.szwajca@amu.edu.pl
* Correspondence: akelarakis@uclan.ac.uk; Tel.: +44-017-724-172

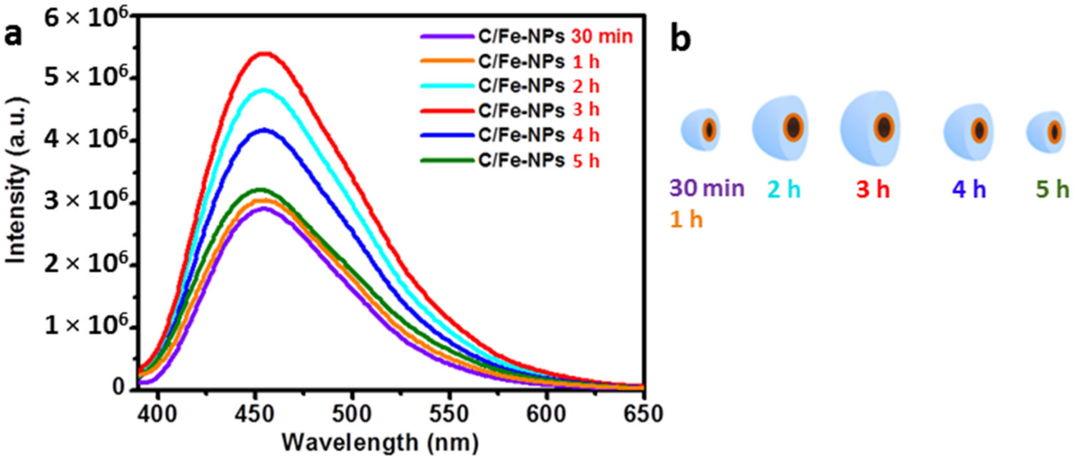

Figure S1. (**a**) The PL spectra (λ_{ex} = 375 nm) of aqueous dispersions of C/Fe-NPs prepared from identical reactant mixtures with C/31Fe-NPs, but at various times of pyrolysis (**b**).

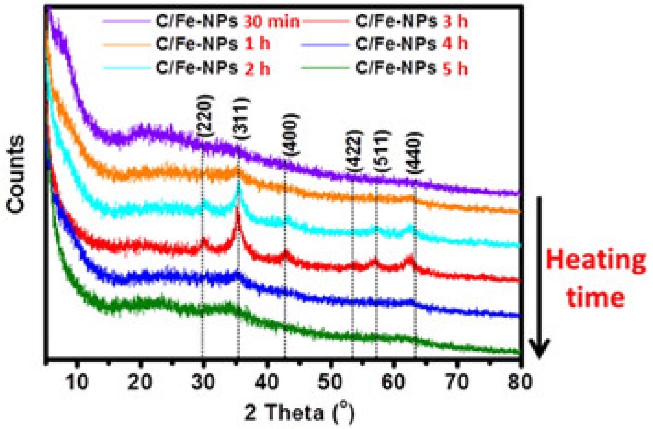

Figure S2. PXRD patterns of C/Fe-NPs prepared from identical reactant mixtures with C/31Fe-NPs but at various times of pyrolysis.

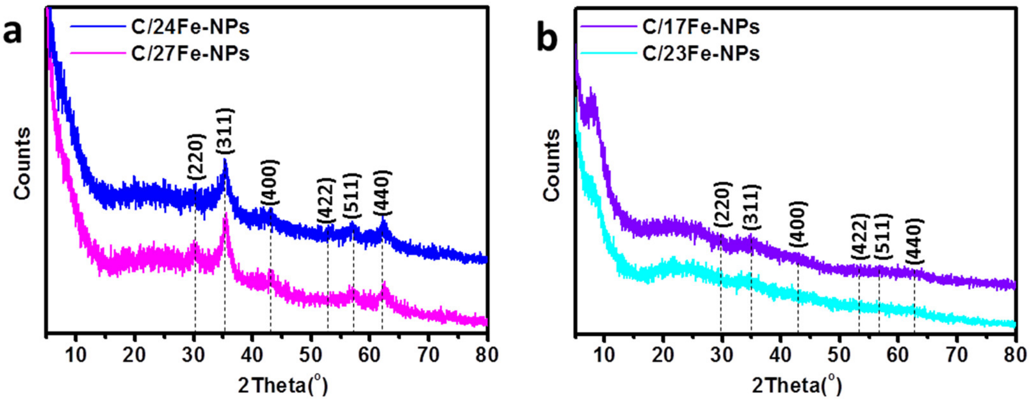

Figure S3. (**a**) PXRD patterns of C/27Fe-NPs (magenta) and C/24Fe-NPs (blue) along with (**b**) PXRD patterns of C/23Fe-NPs (cyan) and C/17Fe-NPs (violet).

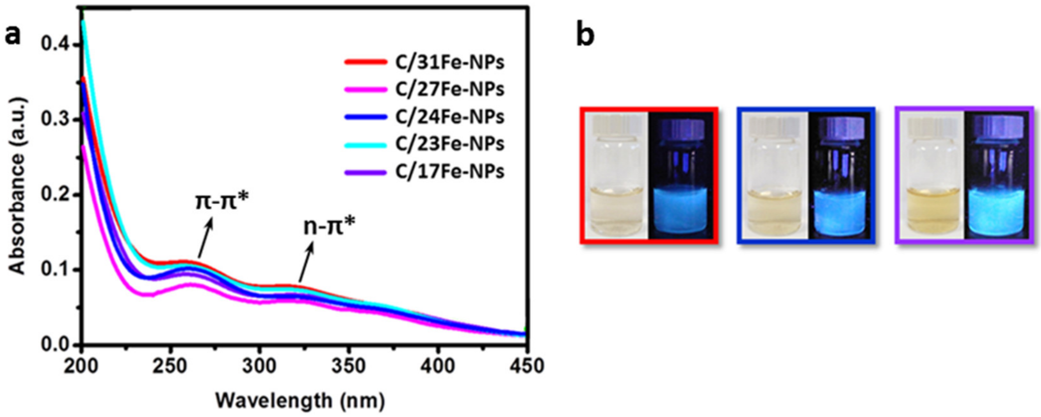

Figure S4. (**a**) Absorption spectra of aqueous dispersions of 0.01 mg/mL C/Fe-NPs; (**b**) Photos of C/31Fe-NPs (red), C/24Fe-NPs (blue) and C/17Fe-NPs (purple) under daylight and ultraviolet light.

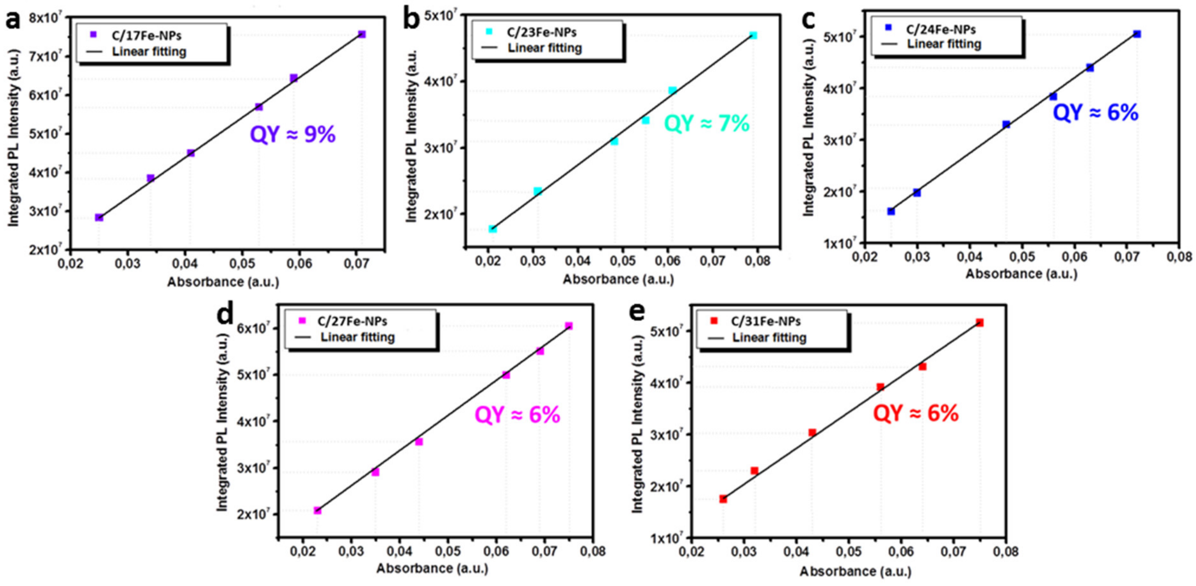

Figure S5. Integrated PL intensity of (**a**) C/17Fe-NPs, (**b**) C/23Fe-NPs, (**c**) C/24Fe-NPs, (**d**) C/27Fe-NPs and (**e**) C/31Fe-NPs in water as a function of optical absorbance at 365 nm.

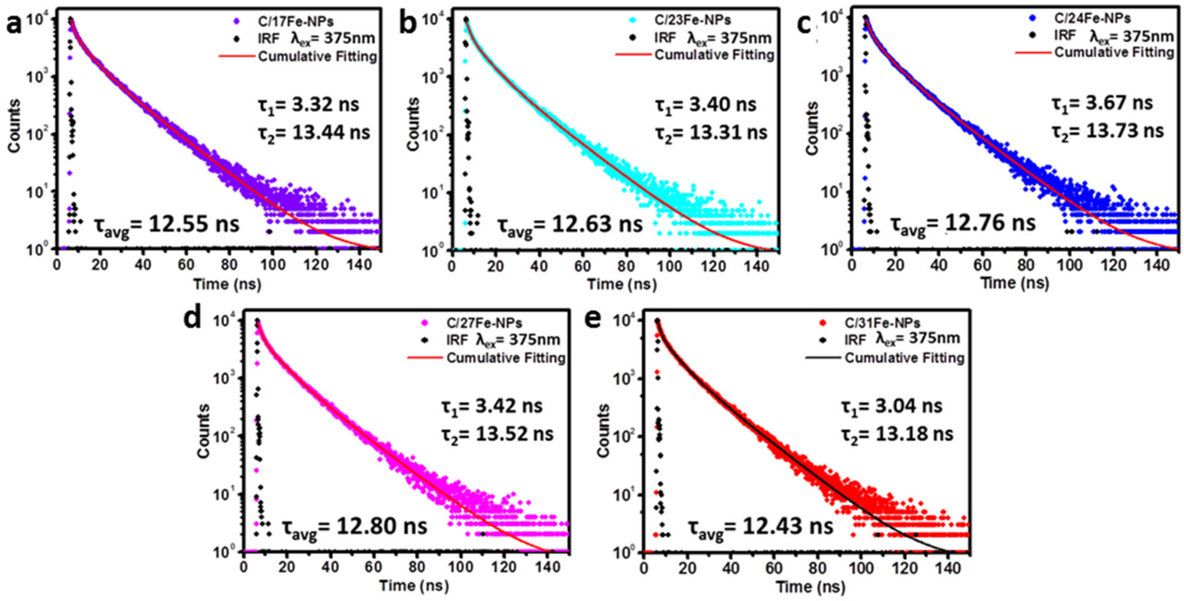

Figure S6. Time-resolved fluorescence decay profiles for aqueous solutions of (**a**) C/17Fe-NPs, (**b**) C/23Fe-NPs, (**c**) C/24Fe-NPs, (**d**) C/27Fe-NPs, (**e**) C/31Fe-NPs (**e**) at λ_{ex}= 375nm.

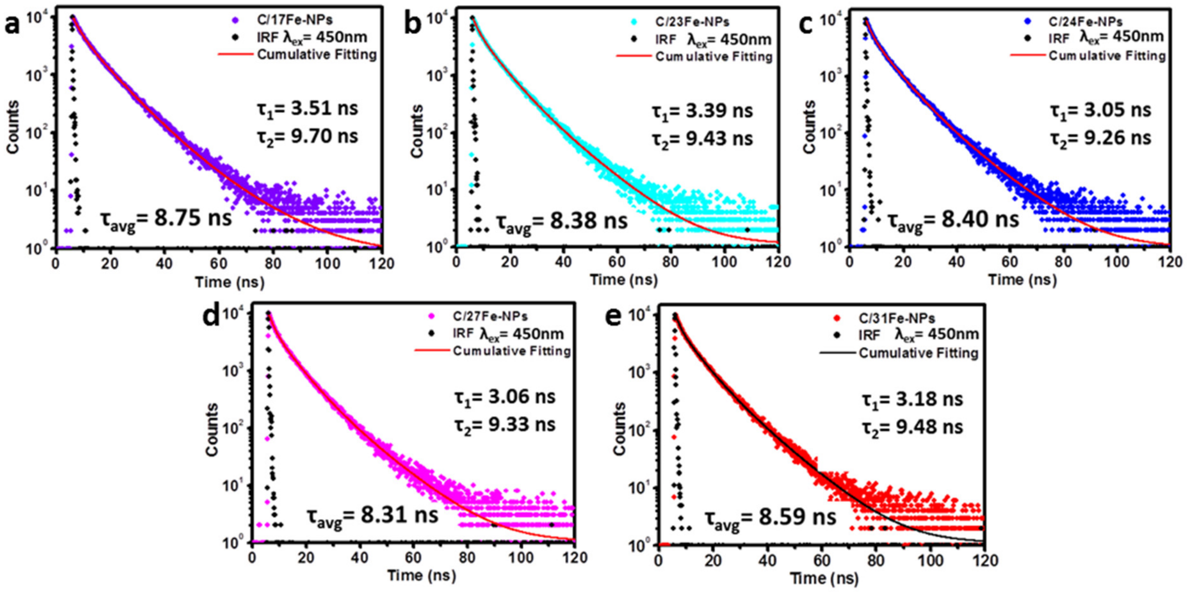

Figure S7. Time-resolved fluorescence decay profiles for aqueous solutions of (**a**) C/17Fe-NPs, (**b**) C/23Fe-NPs, (**c**) C/24Fe-NPs, (**d**) C/27Fe-NPs, (**e**) C/31Fe-NPs at λ_{ex}= 450 nm.

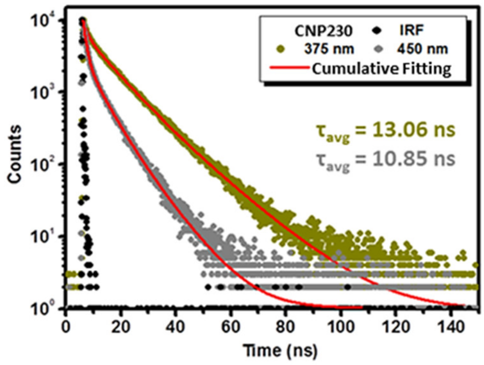

Figure S8. Time-resolved fluorescence decay profiles for aqueous solutions of CNP230 recorded at λ_{ex}= 375 nm (grey colour) and λ_{ex}= 450 nm (dark yellow colour).

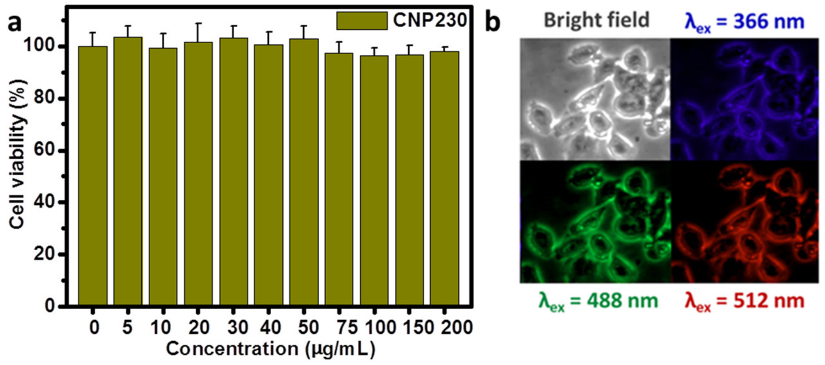

Figure S9. (**a**) The MTT assay results for HeLa cells incubated with CNP230 for 24 h; (**b**) The fluorescence microscope images of HeLa cells with internalized CNP230.
